# Cognitive remediation versus active computer control in bipolar disorder with psychosis: study protocol for a randomized controlled trial

**DOI:** 10.1186/s13063-016-1275-7

**Published:** 2016-03-12

**Authors:** Kathryn Eve Lewandowski, Sarah H. Sperry, Dost Ongur, Bruce M. Cohen, Lesley A. Norris, Matcheri S. Keshavan

**Affiliations:** McLean Hospital/Harvard Medical School, 115 Mill St., Belmont, MA 02478 USA; Beth Israel Deaconess Medical Center/Harvard Medical School, 75 Fenwood Rd., Boston, MA 02115 USA

**Keywords:** Bipolar disorder, Cognitive, Remediation, Cognitive training, Trial, Control

## Abstract

**Background:**

Cognitive dysfunction is a major feature of bipolar disorder with psychosis and is strongly associated with functional outcomes. Computer-based cognitive remediation has shown promise in improving cognition in patients with schizophrenia. However, despite similar neurocognitive deficits between patients with schizophrenia and bipolar disorder, few studies have extended neuroscience-based cognitive remediation programs to this population.

**Methods/Design:**

The Treatment to Enhance Cognition in Bipolar Disorder study is an investigator-initiated, parallel group, randomized, blinded clinical trial of an Internet-based cognitive remediation protocol for patients with bipolar disorder I with psychosis (*n* = 100). We also describe the development of our dose-matched active control paradigm. Both conditions involve 70 sessions of computer-based activities over 24 weeks. The control intervention was developed to mirror the treatment condition in dose and format but without the neuroplasticity-based task design and structure. All participants undergo neuropsychological and clinical assessment at baseline, after approximately 25 hours of study activities, post treatment, and after 6 months of no study contact to assess durability. Neuroimaging at baseline and post treatment are offered in an “opt-in” format. The primary outcomes are scores on the MATRICS battery; secondary and exploratory outcomes include measures of clinical symptoms, community functioning, and neuroimaging changes. Associations between change in cognitive measures and change in community functioning will be assessed. Baseline predictors of treatment response will be examined.

**Discussion:**

The present study is the first we are aware of to implement an Internet-based cognitive remediation program in patients with bipolar disorder with psychosis and to develop a comparable web-based control paradigm. The mixed online and study-site format allows accessible treatment while providing weekly staff contact and bridging. Based on user-provided feedback, participant blinding is feasible.

**Trial registration:**

ClinicalTrials.gov NCT01470781; 11 July 2011.

## Background

Cognitive impairment is common in patients with bipolar disorder (BD) and is associated with poorer functional outcomes [1–5] and longer time to recovery after a first episode [6]. Over time, increasing illness burden appears to be associated with increased neurocognitive dysfunction [7]. Despite strong associations between cognitive impairment and functional outcomes, treatment for these symptoms is inadequate. Medications produce modest overall improvement in cognitive symptoms, and may even worsen some aspects of cognition [8–11]. As few as one third of patients achieve functional recovery over time [12]. At least partial disability is reported in approximately 80 % of patients with BD; as many as 65 % of patients report being unemployed even after clinical recovery, and patients experience significant disability in daily living and social functioning [13]. As cognition is among the strongest predictors of functional outcomes (e.g., [3, 4]), and consistent with a European College of Neuropsychopharmacology expert report [13], it is critical to target this key symptom dimension in patients with BD [1, 4, 12, 13].

### Cognitive deficits in bipolar disorder

Patients with BD exhibit deficits in memory, executive function, and processing speed/efficiency that persist during euthymic phases and over time [14–18]; patients with BD with a history of psychosis (BDP) may be especially cognitively impaired [14, 19, 20]. Cognitive remediation (CR) is a promising treatment approach addressing neurocognitive dysfunction in the hopes that improved cognitive performance will translate to better community outcomes. A meta-analysis of 40 studies of CR for adults with schizophrenia (SZ) found that these programs produced moderate, durable effects on cognitive functioning [21]. A recent meta-analysis of 16 studies reported moderate effects of CR on cognition in affective illness (primarily affective psychosis) similar to findings in SZ [22]. Despite the substantial overlap in cognitive impairments between SZ and BD – qualitatively and in some studies quantitatively – only one study to date has reported outcomes after CR in patients with BD. In a 14-session cognitive training program focusing on neurocognitive deficits and residual symptoms of depression in patients with BD, CR was associated with improved executive functioning, which was related to improved vocational performance [23].

### Challenges in cognitive remediation

Typical CR paradigms require multiple training sessions weekly, which may be prohibitive for potential study participants. Many of the CR programs that have shown efficacy in patients with SZ have studied chronic patients who are able to attend sessions at community-based programs multiple times per week, due to high levels of disability and low instrumental role involvement (e.g., [24]). While patients with BD exhibit considerable disability, especially compared to premorbid functioning, patients with SZ or schizoaffective disorder (SZA) exhibit greater morbidity and disability on average [25], indicating that treatments requiring multiple weekly sessions at study sites may be unrealistic, especially for patients who are even partially engaged in work or school roles. With rapid advancements in online cognitive training programs, there is considerable potential for Internet-based treatments to improve access to training for patients. However, reports suggest that face-to-face “bridging” sessions, which create an opportunity for subjects to explicitly consider implementation of newly-developed cognitive skills in daily life, are important for generalizing the effect of CR treatments to daily functioning [26]. Thus, a tension exists between reducing participant burden through remote access and conducting treatment delivery in the most effective manner.

Inclusion of adequate comparison conditions is challenging in CR paradigms given the active nature of the treatment. “Placebo” conditions are particularly challenging with these types of programs, as participants are keenly aware of the elements of participation throughout the program. The majority of projects that have included an active control group have selected individual or group rehabilitative or therapeutic activities such as Enriched Supportive Therapy [27], supportive group therapy [28], cognitive behavioral therapy (CBT) [29], Integrated Psychological Therapy [30], treatment as usual plus group activities [31], vocational rehabilitation, occupational therapy, supported employment [32–34], computer skills training plus day treatment [35] or social skills training [36]. These types of comparison conditions allow for the control of hours of study staff contact and other factors associated with involvement/adherence to a psychosocial intervention; however, they do not control for other aspects of the CR intervention such as computer interaction or game engagement, and they do not reasonably permit double-blindness – a particular challenge for interpretation of results as poor masking of treatment allocation in clinical trials has been shown to inflate treatment effects [21].

Recently several game-format comparison conditions have been developed for CR paradigms attempting to match the computer/gaming elements of CR but with nonspecific content; these include computer games or action videogames [24, 37–39], some paired with additional group or coaching activities to match for other elements of the active treatment condition [37, 38]. No studies to date have published findings including Internet-based controls that can be overseen via remote access; however, as Internet-based CR programs are in active development and use, comparable Internet-based paradigms that will permit active comparison are needed.

CR programs vary considerably in terms of methodology, and it is not clear which if any aspect(s) of treatment design influence outcomes. Variables to consider include total duration of treatment, number of sessions, treatment intensity (i.e., “density” of sessions), drill and practice approaches versus strategy use, and individual versus group format, among many other characteristics. A meta-analysis of 40 CR studies did not find any effect of treatment characteristics on cognitive outcomes [21], although programs that included strategy use showed a trend toward greater functional improvements [21, 40].

Finally, it is possible that participant characteristics may influence treatment response. In their meta-analysis, Wykes and colleagues [21] found no effect of most baseline characteristics including age, baseline cognitive functioning, or chlorpromazine equivalents on outcomes after CR; however, lower clinical symptoms at baseline were associated with greater effects of treatment on cognitive outcomes.

In this report we aimed to (1) describe our Internet-based neuroplasticity-informed CR protocol for BDP, (2) describe the development of our Internet-based, dose-matched active control, and (3) assess the ability of our study design to remain blinded.

## Methods/Design

This study and all associated procedures comply with the ethical standards of the relevant national and institutional committees on human experimentation and with the Helsinki Declaration of 1975, as revised in 2008, and have been approved by the McLean Hospital Institutional Review Board. This project is registered with ClinicalTrials.gov (NC01470781), and all procedures and reporting of our primary and secondary outcomes will follow Consolidated Standards of Reporting Trials (CONSORT) 2010 guidelines [41].

### Participants

**Fig. 1:**
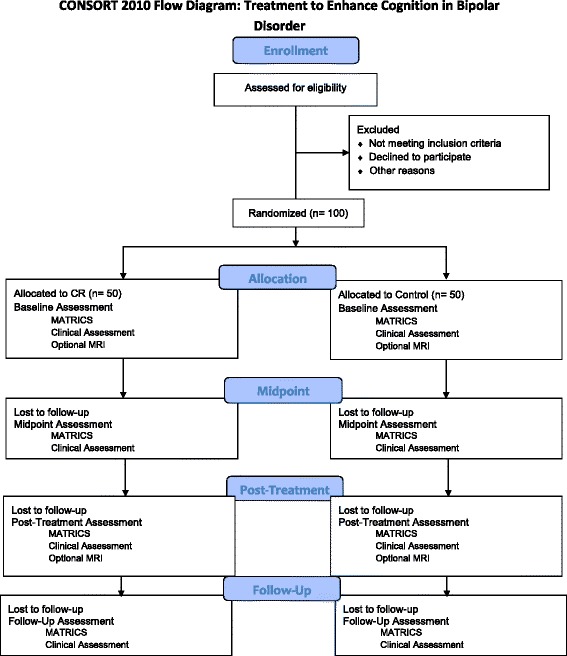
Consolidated Standards of Reporting Trials (CONSORT) 2010 flow diagram: Treatment to Enhance Cognition in Bipolar Disorder Allocation and study flow

Outpatients with idiopathic BD Type I with a history of psychosis between the ages of 18 and 50 (*n =* 100) are recruited through the McLean Hospital Psychotic Disorders Program and fliers posted at the hospital (See Fig. 1). All participants are informed of the study procedures, including randomization to either treatment or control conditions, and provide written informed consent. All patients are symptomatically stable outpatients at the time of enrollment, and have been out of the hospital for at least 1 month with Positive and Negative Syndrome Scale (PANSS) scores under 75, ratings of 3 (mild) or less on the PANSS psychosis items, and Young Mania Rating Scale (YMRS) scores below 6. We set these criteria based on findings that baseline clinical symptoms are associated with treatment response [21]. To reduce medication confounds, only patients on monotherapy lithium or valproate plus an atypical antipsychotic (excluding clozapine) are eligible. Patients taking anticholinergic medications are excluded, as they produce cognitive dysfunction and may interfere with CR response [42, 43]. Exclusion criteria include: substance abuse in the past month; substance dependence in the past year; history of seizure disorder or head injury with loss of consciousness; anticholinergic medications (e.g., benztropine), clozapine, and topiramate.

### Power calculation

For the total sample, we aim to recruit 100 participants, which will yield approximately 50 participants per group. This sample size is based on power calculations using a conservative estimate of effect size of two thirds of the effects on cognition of published findings using similar duration and paradigm CR in patients with SZ (*d* = 0.74–0.89) [24, 44] and anticipating attrition at approximately 15 % [44]. One-tailed alpha for sample-size estimates was selected because directional effects are hypothesized (CR greater than control intervention) and have been established in other populations. Thus, with alpha at 0.05 we have 80 % power to detect effects of 0.54 or greater.

### Assessment measures

#### Cognition

Cognitive functioning was assessed using the battery developed by the Measurement and Treatment Research to Improve Cognition in Schizophrenia (MATRICS) initiative [45]. While the MATRICS Consensus Cognitive Battery (MCCB) was developed for cognitive assessment in SZ, the MCCB has been validated in patients with BD [46, 47]. The MATRICS battery includes 10 tasks that measure processing speed (Brief Assessment of Cognition in Schizophrenia Symbol Coding, Animal Fluency, Trails A), attention (Continuous Performance Test), working memory (WMS-III Spatial Span, Letter-Number Span), verbal learning (Hopkins Verbal Learning Test – Revised), visual learning (Brief Visuospatial Memory Test – Revised), problem solving (Neuropsychological Assessment Battery Mazes) and social cognition (Mayer-Salovey-Caruso Emotional Intelligence Test). Total administration time is 60–90 minutes.

#### Clinical and community functioning

Clinical and community functioning assessment included: the Positive and Negative Syndrome Scale (PANSS; [48]); Young Mania Rating Scale (YMRS; [49]); Montgomery-Asberg Depression Rating Scale (MADRS; [50]); Multnomah Community Ability Scale (MCAS; [51]); Social and Occupational Functioning Assessment Scale (SOFAS; [52]). The MCAS measures functioning in psychiatric patients in multiple domains (e.g., social effectiveness, independence, meaningful activities). The SOFAS is a 100-point scale similar to the Global Assessment of Functioning that evaluates social and occupational functioning not directly influenced by symptom severity.

#### Feedback survey

A user feedback survey was created by our group to evaluate participants’ experiences using the CR or control activities, and to evaluate participants’ beliefs about their study assignment to test the effectiveness of the blind. The first 13 items ask about tolerability, including enjoyment of the tasks, especially compared to other types of treatment, perception of difficulty, stressfulness of the activities, feelings of competency generated by participation, and whether users would continue to participate if given the option. Responses are given using a Likert scale (1–5) with higher scores indicating stronger response; 4 of the 13 items inquired about negative experiences such that higher scores were associated with more negative responses; these items were reverse scored so that in all cases a higher score indicates more positive response. Cumulative scores range from 13 to 65. Subjects were also asked in a forced-choice item to indicate which treatment condition they believed they were assigned to, and then asked to rate the certainty of their selection.

#### Additional outcome measures

We are also collecting neuroimaging data and measures of reward processing at baseline and post treatment in order to begin to examine baseline predictors of treatment response and mechanisms of action of the treatment. These procedures are offered in an “opt-in” format, meaning that participants do not have to consent to (or be eligible for) neuroimaging or reward processing tasks in order to participate in the CR trial.

### CR treatment

Given that this was the first extension of computer-based, drill and practice CR to patients with BDP, and the considerable overlap in terms of cognitive dysfunction in SZ and BDP, we selected a CR paradigm that has demonstrated efficacy in patients with SZ [24]. We selected a dose that – based on previous findings – was likely to be adequate to demonstrate cognitive effects, so that failure of the treatment would not likely be due to under-dosing. Active treatment involved 70 hours of computer-based activities at an intensity of three sessions per week using the BrainWorks programs by PositScience. Games were developed based on a recovery model of neural plasticity [53]. Many cognitive deficits (e.g., memory) are believed to be secondary to more core processing issues, including slow processing speed and poor encoding of sensory stimuli. Thus, these programs target root causes of abnormalities in brain function using a “bottom-up” approach, training sensory processing early in the program and adding “higher-order” tasks as the activities progress. Programs are designed to improve processing speed and efficiency (e.g., quick and accurate stimulus identification), reaction time, attention, concentration, and working memory. Programs self-adjust based on user performance to keep participants working at 80 % proficiency.

The CR paradigm is accessed online through a web portal created by BrainWorks, which allows study participants to engage with the program from any computer with Internet access. In order to reduce travel burden and allow greater flexibility for participants to complete the sessions they are allowed to complete two of the three weekly sessions remotely; one weekly session is to be completed with the study staff at the study site in order to provide coaching and bridging opportunities. Study administrators track participant adherence remotely through an administrator account, which allows staff to check when participants logged onto their programs and what they did while they were there.

### Computer control

We developed a computer-based control that mirrored the CR program in number of training sessions, online format, administrator contact, and general activity format so as to control for as many nonspecific effects of the CR treatment as possible. However, the control does not include tasks that should substantially address the cognitive deficits typical of BDP. Thus, the control condition involves engagement in active computer games, but without the step-wise, “bottom-up” approach or adaptive nature of the tasks. In order to monitor adherence to the protocol – a particular concern given the remote access to games offered in the CR condition – it was necessary to develop a comparison condition that also permitted study staff to track remote usage during at-home sessions. Sessions were created using generic games administered via the online interface Sporcle (www.Sporcle.com), a collection of thousands of quiz-type activities. We carefully selected several hundred games to include a broad array of activities that most people have knowledge of. Games included identification activities (e.g., identify pictured fruits and vegetables), content-based activities (e.g., label states on a map), and some timed activities (e.g., complete as many basic arithmetic problems as possible in 5 minutes). Data from thousands of users documenting accuracy rates for each quiz are freely available online; we used these data to rate each quiz for difficulty based on total quiz completion/accuracy rates. This was undertaken to ensure that games were not overly difficult. Games were excluded if they had an accuracy rate of less than 40 %. Average accuracy for selected quizzes was approximately 73 %.

Similar to the CR condition, an administrator account allows study staff to track subject activity via a social networking-style format that allows the administrator to request that participants allow themselves to be followed online. Through this process the study administrators are able to determine when participants logged in, which games they engaged in, and what their response accuracy was. In order to maintain confidentiality all subjects are assigned a study-based user identity (ID) that allows the administrator to identify individual participants without revealing any personal information.

Games were divided into 70 sessions of prescribed activities to mirror the format and length of the treatment condition. Types of activities were distributed so that no single session included only one type of game (e.g., all rapid-response games). Participants are given “prescription cards” each week listing the games they are to engage in during each weekly session. As in the CR condition, participants may elect to play two of the three weekly sessions remotely via web access; one weekly session is completed at the study site with staff.

### Procedures

Randomization was started using a simple randomization approach, but due to increasing imbalance this approach was switched to a blocked random design with a block size of 10. The Research Randomizer tool (www.Randomizer.org) was used to generate the random allocation sequence. The principal investigator (PI), KEL, generated the sequence, which was given to enrollment staff who are responsible for group assignment. We employed a parallel design with an allocation ratio of 1:1. The randomization key is kept by a single staff member, who provides the treatment and/or communicates assignment information directly to treatment staff. Assessment staff are not informed of group assignment (see below). Participants are not informed of their group assignment. During the consent process participants are explicitly informed that participation in the study involves random assignment to either the treatment condition or an active control, that they may be assigned to the control condition, and that they will not be informed of their group assignment. Participants are informed that they may request knowledge of their study assignment at the end of the follow-up period.

In this study, both patients and assessment staff remain blind to group membership through completion of the follow-up assessment; however, treatment administrators are not able to remain blind to group assignment. Treatment administrators meet with participants weekly to discuss their progress and one session weekly is completed on site with treatment staff; thus, treatment administrators are not able to remain blinded to group. We attempt to maintain the participant and assessment staff blind in several ways: enrollment staff and treatment administrators never conduct assessments at any time point; the assessment staff are not located in the same office space as the treatment administrators, and do not observe or participate in weekly sessions; participants are reminded that assessment staff cannot know which group they were assigned to and it is requested that any mention of the activities be avoided during assessments; subject IDs and enrollment dates are not included in the database accessible to staff who are blind to group membership, and non-sequential dummy codes are created for the purposes of data access by blinded staff members. The feedback survey described above contains two questions regarding participants’ beliefs about group membership, which aim to assess which group participants believe they were in, and their certainty about this belief. These data are used to help us determine whether or not we were able to maintain the blind.

Both conditions involve 70 sessions (3 per week for 24 weeks). All participants attend one session per week at the study site; two sessions are completed remotely via Internet login. Weekly in-person sessions include an update (CR and control) and a bridging session (CR only), plus one CR or control session with study staff. Study staff are able to track user progress and adherence via an administrator account, and send reminder calls or emails upon request or if participants do not complete their at-home sessions in a timely fashion.

Assessments are conducted at baseline, midpoint (after approximately 25 hours of training) post treatment (within 1 week of completing the treatment/control), and at follow-up (6 months after completion of the treatment/control). All subjects complete clinical and cognitive assessments; neuroimaging is presented as an “opt in” portion of the study. Clinical and cognitive assessments are typically conducted in the same session. Baseline assessments occur 2 weeks or less prior to the start of training; post-treatment assessments are conducted within 1 week of completing the final training session.

### Statistical approach

Our primary aim is to evaluate the effect of CR on cognition in patients with BDP compared to a computer-based control. The primary outcome measures of interest are MCCB scores. Primary analyses will be based on composite scores; secondary analyses will examine domain scores if composite findings are significant. The main analysis will be an intent-to-treat analysis, which includes all randomized subjects regardless of compliance to reduce potential for post-randomization bias and inflation of Type I errors. We will use a linear mixed effects model; inclusion of random effects accounts for the correlation among repeated measures. Outcome measures will include the three post-baseline measures of cognition; the baseline measure will be adjusted for by including it as a covariate. The test of no treatment difference will be based on a joint test of the group and group-by-time interaction.

The secondary aim is the examination of potential mechanisms for changes in community functioning. It is hypothesized that cognitive (but not clinical) change will mediate changes in community functioning. Thus, community functioning will be modeled adjusting for composite neuropsychological (or clinical) change using linear mixed effects models to relate changes in community functioning to treatment group while adjusting for differences in cognitive (or clinical) change.

## Discussion

The present study is the first we are aware of to implement an Internet-based CR paradigm in patients with BDP and to develop a comparable Internet-based control. The hybrid online/study site format allows accessible treatment while providing opportunities for staff contact and bridging. Our newly developed web-based control is comparable to the treatment in terms of administration and interface: it is dose-matched, including requirements for in-person visits and study staff contact; administrator oversight permits adherence monitoring; sessions are similar in format to those of the CR paradigm.

Based on preliminary feedback from our feedback survey, participant blindness can be successfully maintained with our present paradigm. Responses from the first 22 participants to complete the survey showed that subjects did not differ in terms of which condition they believed they were randomized to: 86 % of subjects in the treatment and 63 % of subjects in the control reported believing that they had participated in the active CR treatment (Chi^2^ = 1.56, *p* = 0.20). On a three-point scale evaluating confidence in their belief of their reported assignment (very (3), somewhat (2), or not at all (1)), participants had low confidence in their ratings in both treatment and control conditions (mean = 1.64, SD = 0.63 and mean = 1.63, SD = 0.92, respectively; *t =* 0.05, *p* = 0.96). Our newly developed active control permits examination of specific elements of our CR paradigm by accounting for many of the nonspecific elements of participation in the study while maintaining the participant and assessment staff blind.

Ultimately, our primary aim is to evaluate efficacy of this CR program compared to active control. We also aim to examine predictors of treatment adherence and response, and neurobiological mechanisms of action using magnetic resonance imaging (MRI) techniques. Better understanding of both predictors of treatment response and the mechanisms of action of CR will allow further refinement and individualization of treatment efforts. We will also be able to examine the role of baseline characteristics, reward processing, and neurobiological response in the relationship between cognitive and functional outcomes.

### Limitations

While extensive efforts will be made to minimize missing data, attrition is a major concern in long-duration CR paradigms. We have implemented several techniques for minimizing attrition including monetary bonuses for reaching session milestones (awarded after 10 sessions, 30 sessions, and 50 sessions, in increasing amounts), regular phone and email contact from study staff reminding subjects to complete sessions at home, weekly in-person visits, and weekly session tracking feedback that displays progress and time to next bonus graphically. However, attrition remains a concern. Thus, for all analyses we will use statistical methods that incorporate partially observed data, and assess the sensitivity of results to different assumptions about the mechanism by which data are missing.

In any clinical trial the potential for bias in recruitment or allocation is a concern. All potential subjects meeting enrollment criteria are offered the opportunity to participate; special efforts will be made to ensure that the enrollment of women and minorities matches the demographics of the region. Allocation is done using a blocked random design based on number sequence, without consideration of participant characteristics. Thus, the main source of bias in this study is likely in non-random characteristics of participants who do or do not adhere to the study procedures. As noted above, we will use statistical methods to minimize the potential for such post-randomization bias.

In terms of masking, we have developed an approach that maximizes our ability to include blinding of participants and study staff; however, as noted above, while assessment staff are able to remain blinded to study assignment, due to the nature of the intervention treatment staff are not. Thus, there is the potential for information about group membership to inadvertently become known to participants or to assessment staff. Because poor masking of treatment allocation is associated with potential inflation of treatment effects [21], the issue of blinding in treatment studies is of key importance. Our post-treatment feedback survey suggests that we have been able to maintain participant blinding, but the potential for a break in the blind remains a limitation.

Patients with BD comprise a highly heterogeneous group, creating the potential for dilution of findings of efficacy within subsamples that are more homogeneous. We attempted to increase homogeneity of our sample by restricting enrollment to patients with a history of psychosis, which has been shown to be associated with cognition, and by imposing age and medication restrictions; however, this may restrict generalizability of our findings to patients with specific illness characteristics. If our findings continue to support the feasibility, tolerability, and ultimately efficacy of CR in patients with BDP, future work may extend this treatment to patients with different baseline characteristics in order to examine the question of generalizability and design protocols targeted to subgroups of patients with BDP.

## Trial status

Patient enrollment began in July 2011. By July 2015 76 participants had been enrolled.

## Abbreviations

BD: bipolar disorder
BDP: bipolar disorder with psychosis
CBT: cognitive behavioral therapy
CONSORT: Consolidated Standards of Reporting Trials
CR: cognitive remediation
MATRICS: Measurement and Treatment Research to Improve Cognition in Schizophrenia
MCAS: Multnomah Community Ability Scale
MCCB: MATRICS Consensus Cognitive Battery
PANSS: Positive and Negative Syndrome Scale
SOFAS: Social and Occupational Functioning Assessment Scale
SZ: schizophrenia
SZA: schizoaffective disorder
YMRS: Young Mania Rating Scale
